# Effects of Sodin 5 on the Proliferation and Migration of Human Glioblastoma Cells

**DOI:** 10.3390/toxins18100441

**Published:** 2026-10-13

**Authors:** Veronica Russo, Sara Ragucci, Miriam Russo, Maria Antonietta Oliva, Nicola Landi, Antimo Di Maro, Antonietta Arcella

**Affiliations:** 1 IRCCS Istituto Neurologico Mediterraneo NEUROMED, Via Atinense 18, 86077 Pozzilli, IS, Italy; veronica.2306@hotmail.it (V.R.); miriamrusso39@gmail.com (M.R.); mariaantonietta.oliva@neuromed.it (M.A.O.); 2 Department of Environmental, Biological and Pharmaceutical Sciences and Technologies (DiSTABiF), University of Campania “Luigi Vanvitelli”, Via Vivaldi 43, 81100 Caserta, CE, Italy; sara.ragucci@unicampania.it (S.R.); nicola.landi@cnr.it (N.L.); 3 Institute of Crystallography, National Research Council, Via Vivaldi 43, 81100 Caserta, CE, Italy

**Keywords:** *Salsola Soda*, cytotoxicity, edible plants, glioblastoma, ribosome-inactivating proteins

## Abstract

**Background**: Glioblastoma (GBM) is the most aggressive primary brain tumor and remains difficult to treat because of its highly invasive behavior and resistance to conventional therapies despite surgery, radiotherapy, and chemotherapy. Sodin 5, a novel type-1 ribosome-inactivating protein (RIP), inhibits protein synthesis and displays cytotoxic activity against several tumor cell types, making it a promising candidate for GBM therapy. **Methods**: The antitumor activity of sodin 5 was evaluated in the U87MG glioblastoma cell line and patient-derived primary GBM cell lines (CG, ZAR, and NULU). Cell viability, migration, and molecular changes were assessed after treatment. Biological activity was also investigated under different pH conditions. Vimentin and phosphorylated ERK (p-ERK) expression were analyzed to investigate the mechanisms underlying sodin 5 activity. **Results**: Sodin 5 significantly reduced cell viability in all GBM cell lines. Treatment also inhibited cell migration, accompanied by decreased vimentin expression, suggesting cytoskeletal remodeling and reduced invasive potential. Furthermore, sodin 5 decreased p-ERK levels, indicating modulation of signaling pathways involved in proliferation and motility. The protein retained biological activity under different pH conditions, supporting its potential efficacy in the heterogeneous GBM microenvironment. **Conclusions**: These findings identify sodin 5 as a promising therapeutic candidate for GBM by targeting multiple processes involved in tumor growth and invasion.

## 1. Introduction

Ribosome-inactivating proteins (RIPs) are rRNA N-β-glycosylases (E.C. 3.2.2.22) that catalyze the removal of a conserved adenine residue from rRNA (A4324 in 28S rRNA from rat ribosomes), thereby causing irreversible inhibition of protein synthesis [1]. These proteins are primarily found in Angiospermae (flowering plants) but have also been identified in bacteria and fungi [2,3].

The target of RIPs is the Sarcin–Ricin Loop (SRL) of the large rRNA subunit, a conserved region essential for the binding of elongation factors during translation [1,4]. Depurination of this region prevents interaction with elongation factors, thereby halting translation [5]. In addition, most RIPs are polynucleotide:adenosine glycosylases (PNAG) able to remove adenines from poly(A), RNA, and DNA [6,7], and they also exhibit DNase activity [8].

These enzymes are classically divided into two main groups, type-1 and type-2 RIPs, based on the presence or absence of a quaternary structure. Type-2 RIPs are heterodimeric proteins (~60 kDa) composed of an A-chain (rRNA N-β-glycosylase activity) and a lectin B-chain able to bind cell surface carbohydrates, facilitating cell entry. Type-1 RIPs lack the B-chain and consist of a single polypeptide (~30 kDa), with enzymatic activity [1,9]. A few exceptions, known as type-3 RIPs, are synthesized as inactive precursors and activated through proteolytic processing [9].

RIPs act as defensive proteins, protecting plants against viral infection [10], fungal pathogens [11], and herbivorous insects [12], and they are also involved in plant stress responses [13]. On the other hand, their ability to specifically depurinate rRNA and halt protein synthesis makes them powerful tools in both plant defense (as biopesticides) [14] and oncological research (as immunotoxins or nanoconstructs that selectively kill cancer cells) [15,16].

RIPs that have been well characterized include: (i) ricin, a type-2 RIP isolated from *Ricinus communis* L. seeds, which has been extensively studied in toxicology and biodefense research [17]; (ii) trichosanthin, a type-1 RIP from *Trichosanthes kirilowii* L. tuber, well-known in traditional Chinese medicine [18]; (iii) pokeweed antiviral protein (PAP), a type-1 RIP isolated from *Phytolacca americana* L. seeds with strong antiviral activity [19]; (iv) gelonin, a type-1 RIP isolated from *Gelonium multiflorum* L. seeds, usually employed in antibody–toxin conjugates for cancer therapy research [20]; and (v) saporin 6, a type-1 RIP isolated from *Saponaria officinalis* L. seeds, widely used in immunotoxin construction [21,22].

Recently, our group isolated a novel type-1 RIP, named sodin 5, from the seeds of *Salsola soda* L. (‘agretti’ in Italian) [23]. This protein is non-glycosylated and highly stable, with a melting temperature (Tm) of approximately 76 °C. It is capable of releasing the β-fragment (a hallmark of RIPs’ enzymatic activity), which can be detected by Endo assay [5] and inhibits protein synthesis in a rabbit reticulocyte lysate system, with an IC_50_ value of 4.83 pM [23]. Notably, this value is about 7.7-fold lower than that of saporin 6 (IC_50_ = 37 pM) [24], indicating higher potency. Furthermore, recent studies highlight that sodin 5 exhibits cytotoxic effects against several tumor cell lines (i.e., HeLa, Caco-2, COLO 320, and HT29), inducing cell death through the apoptotic pathway [23], and possesses antifungal activity [23,25].

Considering the above, it is of interest to assess the effects of sodin 5 on glioblastoma (GBM), the most severe and common brain tumor affecting adults. GBM is malignant, infiltrative, expansive, and exhibits rapid growth [26]. These characteristics, together with high angiogenesis, cellular heterogeneity, and the presence of a specific population of brain tumor stem cells capable of proliferating and generating neoplastic glial cells [27], contribute to a poor prognosis with a median survival for this type of tumor of 14 months and a 5-year survival rate of 2% [28,29]. Despite the numerous histopathological variants of GBM, some characteristics common to all types of GBM are cellular and nuclear pleomorphism, microvascular proliferation, and necrosis. Furthermore, GBM cells have the ability to activate numerous resistance mechanisms, which complicate the search for an effective therapy for this tumor. Among the mechanisms of GBM drug resistance to the alkylating agent (temozolomide), the most common is O6-methylguanine methyltransferase (MGMT), a protein specific to DNA repair, whose expression is variable due to acquired methylation of the gene promoter during gliomagenesis. Despite extensive surgical removal of what appears to be all macroscopic disease, both at initial diagnosis and at the time of recurrence, all patients will continue to show tumor growth and progression due to the rapid proliferation of infiltrative disease that persists after surgery. The current standard of care for newly diagnosed disease involves maximal safe resection, followed by six weeks of daily concurrent radiotherapy and chemotherapy with temozolomide (TMZ) [30].

Given the pronounced therapeutic resistance and molecular heterogeneity of glioblastoma, agents capable of exploiting vulnerabilities distinct from those targeted by current standard-of-care treatments may provide valuable new avenues for investigation. The ability of sodin 5 to enter cancer cells, reach ribosomes, induce rRNA depurination, inhibit protein synthesis, and promote apoptotic cell death suggests that this type-1 ribosome-inactivating protein may represent a mechanistically distinct approach to targeting GBM cells. However, the effects of sodin 5 on glioblastoma cells and the molecular mechanisms underlying its potential antitumor activity remain insufficiently characterized. In particular, it is important to determine whether sodin 5 can selectively impair GBM cell viability and whether its cytotoxic effects are associated with ribosomal damage, translational inhibition, and activation of apoptosis. We hypothesize that sodin 5 exerts cytotoxic effects against glioblastoma cells by inducing rRNA depurination and consequent ribotoxic stress, leading to inhibition of protein synthesis and activation of apoptotic cell-death pathways. We further hypothesize that these effects may occur at biologically relevant concentrations and may differ between malignant and non-malignant cells. The overall aim of this study is to investigate the antitumor potential and mechanism of action of sodin 5 in glioblastoma. Specifically, the study will: determine the effects of sodin 5 on the viability and proliferation of GBM cells; characterize whether sodin 5 induces apoptosis and other markers of cellular stress; investigate the molecular pathways associated with sodin 5-induced cytotoxicity; and evaluate the relative sensitivity of GBM cells compared with appropriate non-malignant cellular controls. Together, these studies will establish whether sodin 5 can target glioblastoma through ribosome-mediated mechanisms and provide a mechanistic basis for evaluating this ribosome-inactivating protein as a potential experimental antitumor agent.

Thus, to get insight into the in vitro cytotoxic effect of sodin 5, it was tested against the commercial GBM tumor cell line U87MG and three human primary cell lines (NULU, ZAR, and CG) established from patients’ gliomas [31]. In this context, primary cultures derived from patient GBM tumor samples represent reliable models for GBM and provide valuable tools for the screening of novel and personalized therapeutic approaches [32]. In addition, considering that the GBM tumor microenvironment is highly heterogeneous and exhibits distinctive chemical features such as acidosis, resulting from a shift towards aerobic glycolysis and lactic acid fermentation (Warburg effect) [33], we evaluated the PNAG activity and structural features by far-UV circular dichroism (CD) of sodin 5 under different acidic pH conditions.

## 2. Results

### 2.1. Sodin 5 Purification

Native sodin 5 was purified from *S. soda* seeds as described previously [23]. For the biological assays, the concentration of sodin 5 was determined using a BCA Protein Assay.

### 2.2. Effect of Sodin 5 on Viability and GBM Cell Proliferation

**Figure 1:**
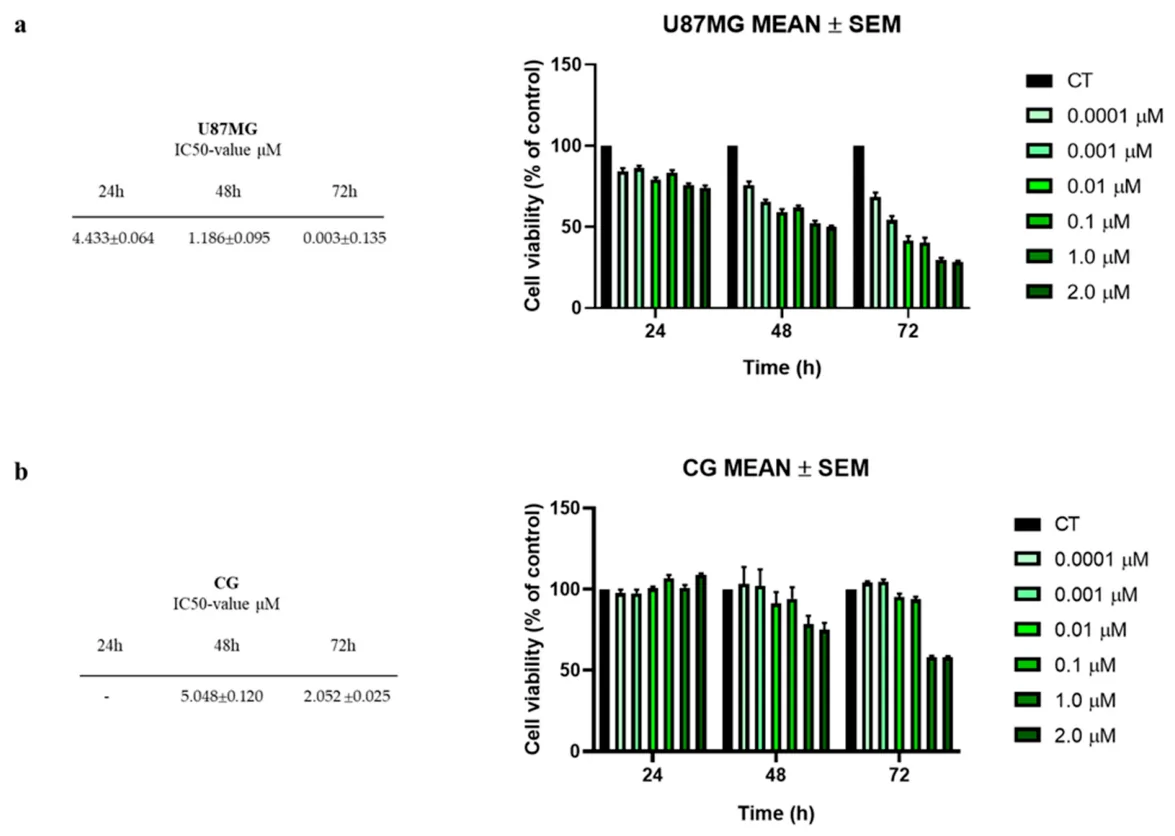
Dose-dependent cytotoxic effect of sodin 5 on the U87MG and CG GBM cell lines. U87MG (**a**) and CG (**b**) IC_50_ values following a single treatment with sodin 5 at concentrations of 0.0001, 0.001, 0.01, 0.1, 1.0, and 2.0 μM at specific time points (24, 48, and 72 h). Untreated cells (CT) were used as a control for comparison of the results and graphical analysis of the three experimental time points (24, 48, and 72 h), expressed as mean ± standard error of the mean (SEM), to evaluate the percentage response of treated cells relative to the control. The black columns represent the untreated controls, while the green columns indicate the treated samples, with a color gradient ranging from light green (lowest concentration) to dark green (highest concentration).

The inhibitory concentration (IC_50_) is a key parameter for understanding the pharmacological properties of a therapeutic agent; this value indicates the concentration of a substance required to reduce cell viability or growth by 50%, thereby providing an important measure of its cytotoxic efficacy. In this specific case, the U87MG and CG cell lines were treated with increasing doses of sodin 5 (0.0001, 0.001, 0.01, 0.1, 1.0, and 2.0 μM) with a single treatment for 24, 48, and 72 h, in order to assess the cytotoxicity of the aforementioned substance as a function of time and concentration. The subsequent MTT assay allowed for the determination of the precise IC50 value at the various experimental time points. As illustrated in Figure 1a, the values obtained for the U87MG line were: 4.333 ± 0.064; 1.186 ± 0.095; and 0.003 ± 0.135 μM at 24, 48, and 72 h, respectively. Figure 1a graphically represents the percentage change over time: all experimental time points are shown within the same bar chart to allow for a direct comparison in assessing the reduction in cell viability of the treated samples compared to the untreated controls. Figure 1b shows the MTT assays of the primary glioblastoma CG cell line, displaying the following IC_50_ values: 5.048 ± 0.120 and 2.052 ± 0.025 µM for 48 and 72 h, respectively. Interestingly, compared to the U87MG cell line, in these primary cells, sodin 5 exhibits an inhibitory effect starting from 48 h of treatment.

**Figure 2:**
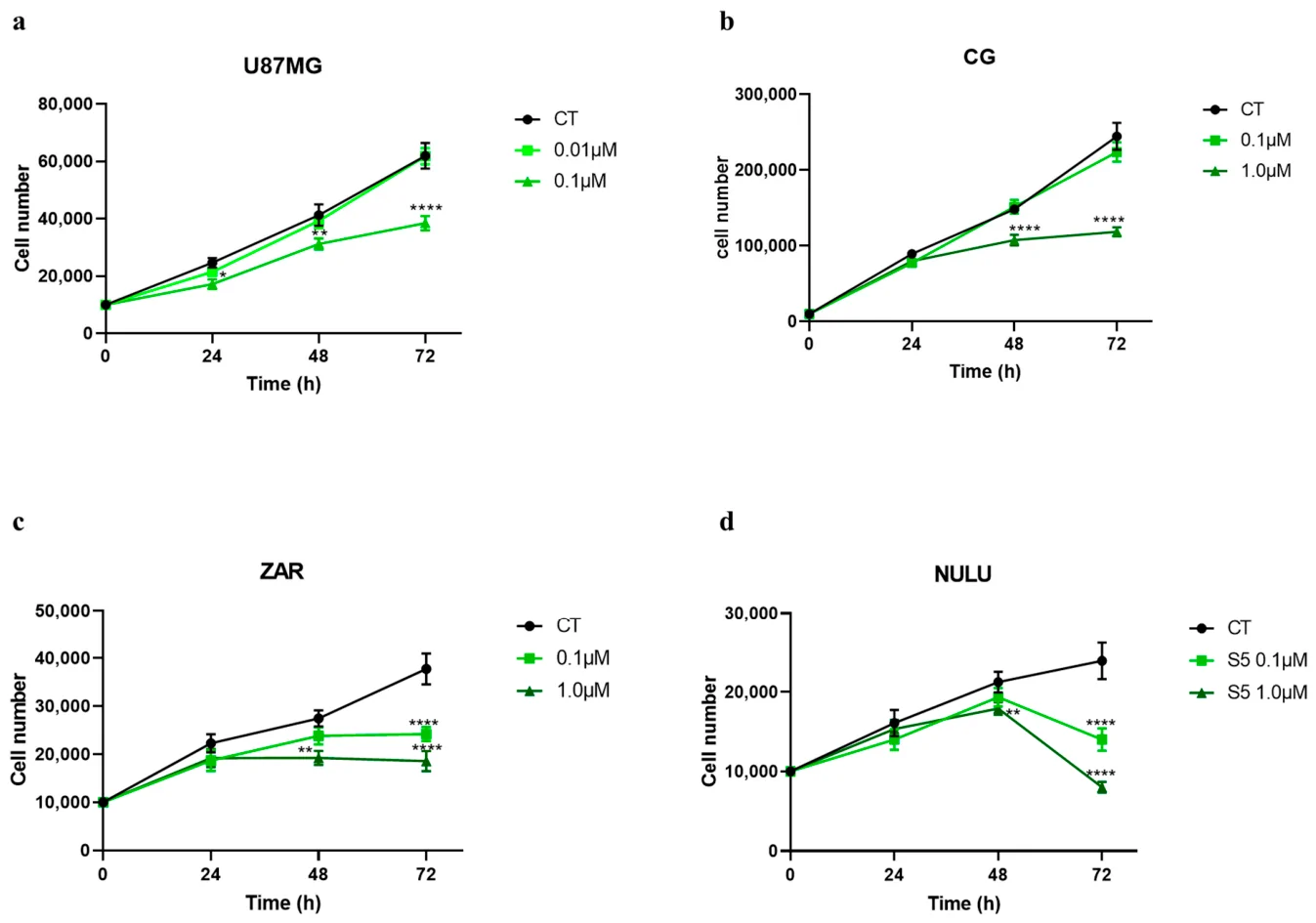
Growth curves in GBM cell lines treated with sodin 5. Graphs show the trend in cell proliferation for the U87MG (**a**), CG (**b**), ZAR (**c**), and NULU (**d**) cell lines. Untreated cells (CT), used as controls, are shown in black, while cells treated with sodin 5 are represented in green with two shades, light green for the lowest protein concentration and dark green for the highest protein concentration, in order to clearly distinguish the different experimental conditions within the graph. Data were processed using GraphPad Prism and are reported as mean ± SEM of three individual experiments (*n* = 3): one-way ANOVA test, Tukey’s multiple comparisons test. According to GraphPad Prism, * *p* values < 0.05, ** *p* value 0.001 to 0.01 (very significant), **** *p* value < 0.0001 (extremely significant).

Based on the IC_50_ values obtained, growth curves were plotted for the various primary cell lines under consideration. As shown in Figure 2, the results revealed a generally similar trend: in U87MG cells, treatment with 0.1 μM sodin 5 resulted in a reduction in growth of approximately 50% at 72 h after induction (Figure 2a).

In the primary CG line, 0.1 μM sodin 5 had no effect on cell growth under the tested conditions, while higher concentrations (1.0 μM) resulted in a reduction of approximately 20% as early as 48 h after treatment, reaching 50% after 72 h (Figure 2b). The primary ZAR line, on the other hand, exhibited a decrease in proliferation similar to the CG line (reduction of 20% and 50% at 48 and 72 h, respectively) when treated with 1.0 μM sodin 5. However, in this specific case, the effect of the substance was also observable at the lower concentrations (Figure 2c). For the primary NULU line (Figure 2d), a marked reduction in cell proliferation was observed for both tested concentrations after 72 h of treatment (approximately 60% and 70%, respectively). The differing response to treatment among these cell lines suggests a variability in sensitivity to sodin 5, likely dependent on the metabolic characteristics of each primary cell type.

**Figure 3:**
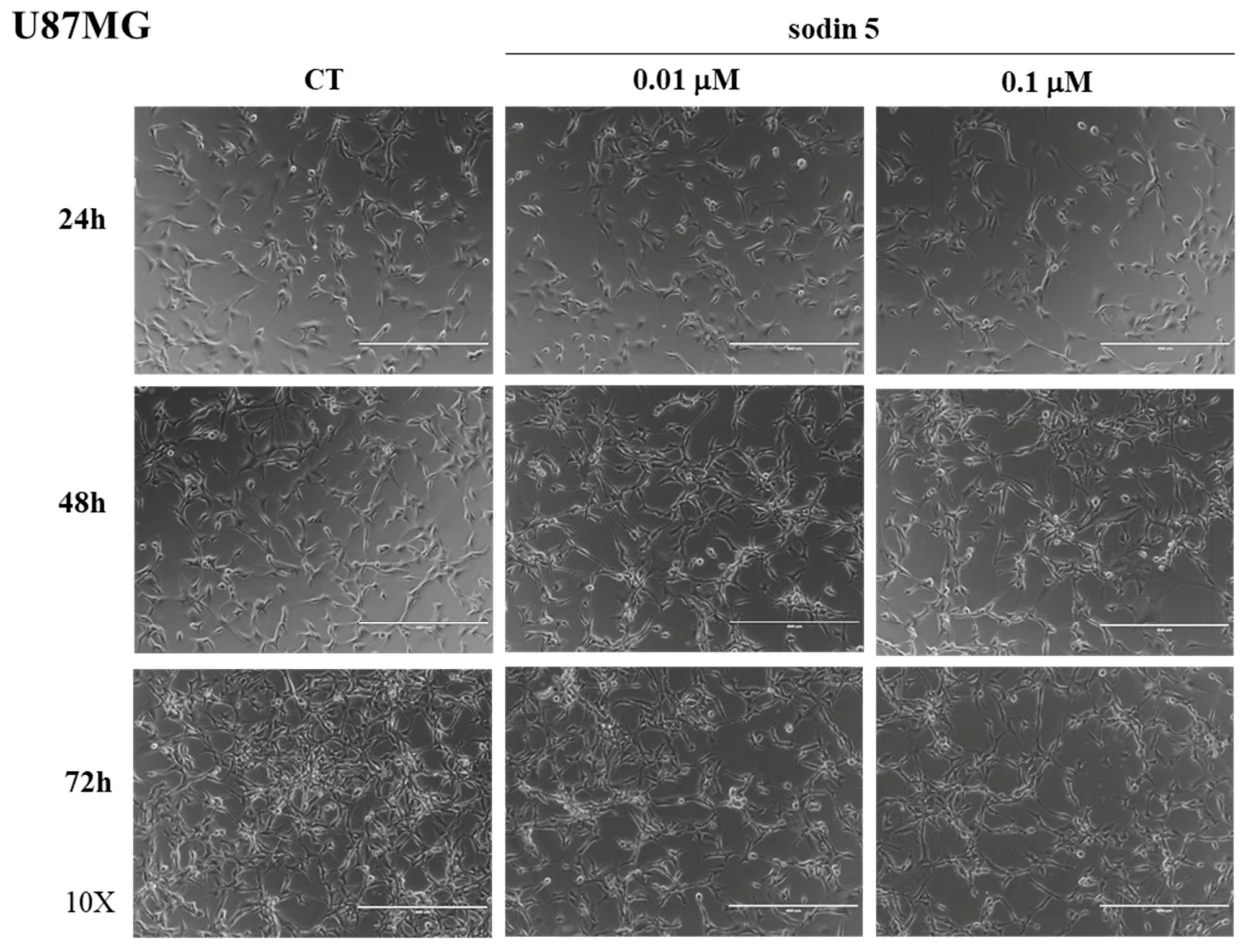
Representative images corresponding to the growth curves of the U87MG continuous GBM cell line treated with 0.01 and 0.1 μM sodin 5 at specific time points. Images were acquired using an EVOS FL microscope at 10× magnification. CT, untreated cells. The scale bar indicates 400 μm; magnification 10×.

Furthermore, the comparison among the different experimental conditions allowed the assessment of the effects of the tested compound on cell morphology and behavior over time. Figure 3 shows representative images of the cell lines analyzed at various time points. After 72 h of treatment, a marked reduction in cell density was observed, consistent with the growth curve data; however, no appreciable morphological alterations were detected in treated cells compared with their corresponding controls.

### 2.3. Effect of Sodin 5 on Migration

**Figure 4:**
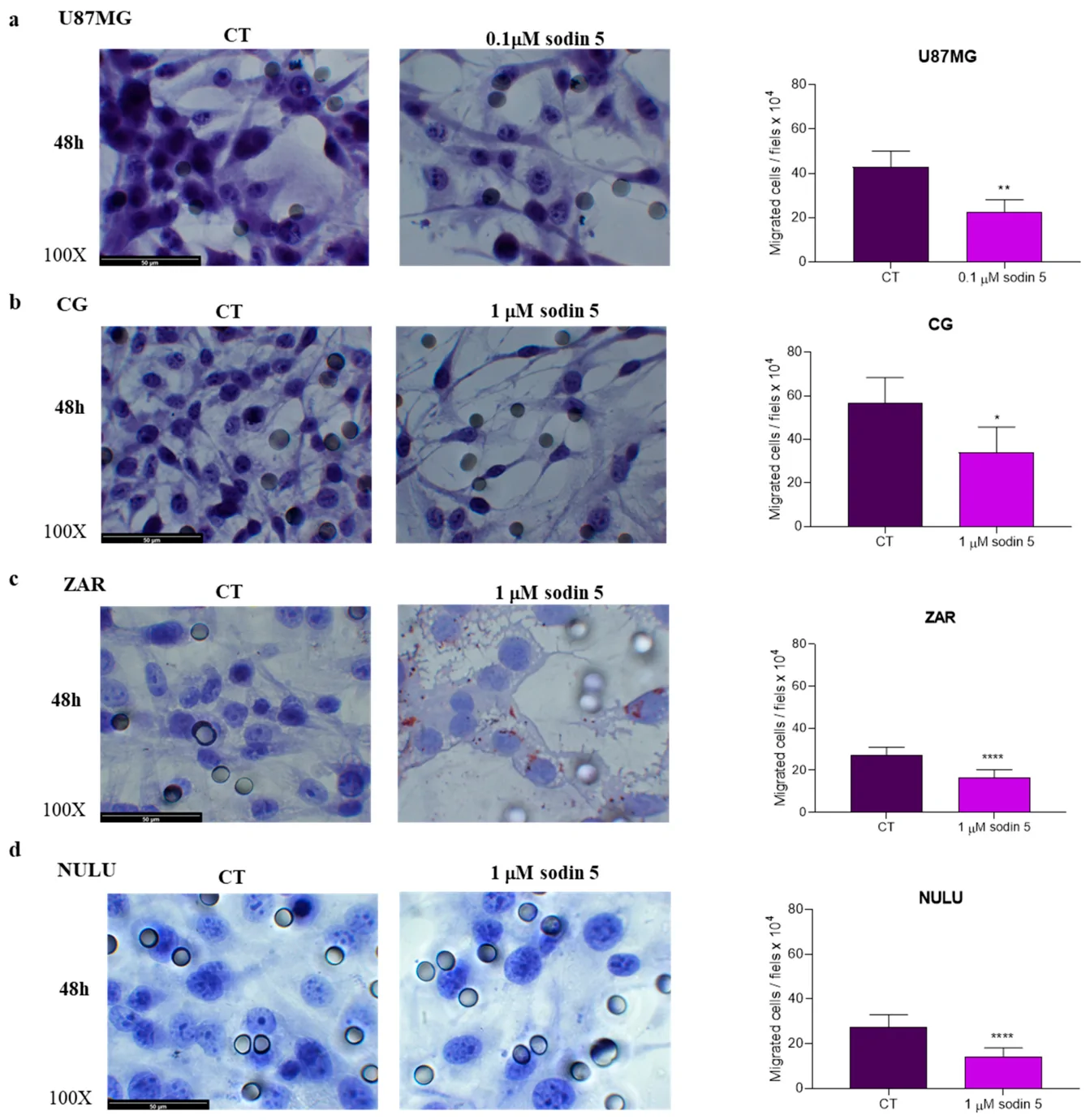
Trans-well migration assay performed on four selected cell lines: the GBM cell line U87MG (**a**) and the three patient-derived primary cell lines, CG (**b**), ZAR (**c**), and NULU (**d**). Cells were treated with sodin 5 at a concentration of 0.1 μM for the U87MG line and 1.0 μM for the primary lines. For each cell line, representative images of both untreated control cells (CT) and treated cells were acquired 48 h post-treatment using optical microscopy. The images were captured using a Zeiss Axiophot 2 microscope at 100× magnification; the scale bar indicates 50 μm. Staining with Cresyl violet was used to visualize and quantify migrated cells. Data were analyzed using GraphPad Prism and are reported as mean ± SEM of three independent experiments (*n* = 3). Statistical significance was determined using an unpaired *t*-test: * *p* values < 0.05; ** *p* value 0.001 to 0.01 (very significant); **** *p* value < 0.0001 (extremely significant).

The Trans-well migration assay was used to determine the effect of sodin 5 on the migration of the continuous GBM cell line (U87MG) and patient-derived primary cell lines (CG, ZAR, and NULU) compared to the untreated control. As shown in Figure 4, all analyzed lines exhibited a reduction in cell migration when subjected to an FBS-induced chemotactic stimulus when treated with sodin 5. Specifically, the percentage of migrating U87MG cells decreased significantly with 0.1 μM sodin 5; 48 h after treatment, the migration rate was approximately 20% compared to 40% in the untreated control (Figure 4a). Similar results were obtained with the primary cell lines used in the experiment; daily induction for 48 h with 1.0 μM sodin 5 resulted in a 20% reduction in the CG cell line (Figure 4b), while ZAR and NULU showed a reduction of approximately 15% (Figure 4c,d) compared to their respective controls.

**Figure 5:**
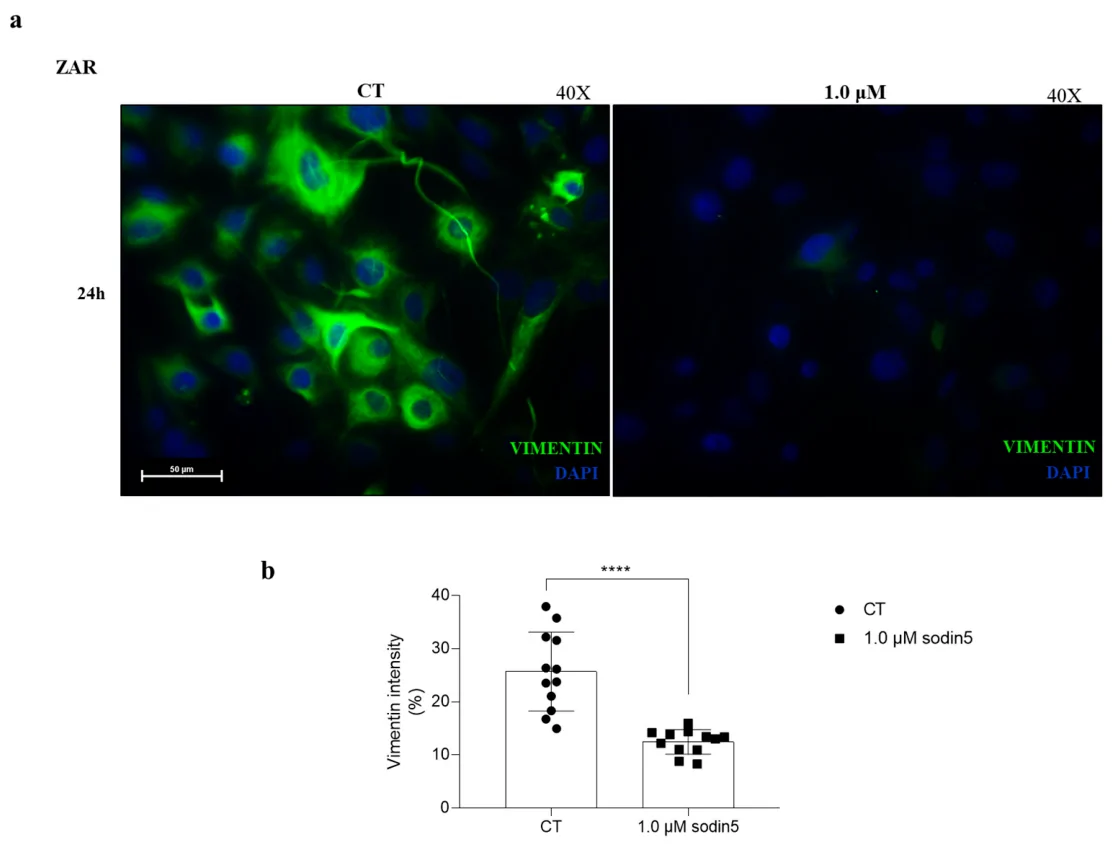
Immunofluorescence analysis of vimentin expression in the ZAR primary cell line. Cells were treated with 1 μM sodin 5 for 24 h. (**a**) Vimentin immunofluorescence is visualized in green, while nuclei are counterstained with DAPI (blue). Representative images were captured using a Zeiss Axiophot 2 microscope at 40× magnification; the scale bar indicates 50 μm (*n* = 3); (**b**) quantitative analysis of vimentin fluorescence intensity (%) using Image J software version 1.53k. Statistical significance: **** *p* value < 0.0001; unpaired *t*-test.

In light of the observed result on cell migration, highlighting the decrease in the number of moving cells in samples treated with sodin 5, vimentin expression was assessed using immunofluorescence in light of the noted effects on cell migration. The latter is a tumor-associated marker involved in cytoskeletal remodeling, as well as in cell migration and invasion into the surrounding microenvironment. Treatment with 1.0 μM sodin 5 resulted in a significant reduction of vimentin fluorescence intensity as early as 24 h post-treatment in ZAR primary cells compared with untreated cells (Figure 5). These findings further support the previously discussed results regarding the inhibitory effect of sodin 5 on cell migration.

### 2.4. Mechanism of Sodin 5 Inhibition

Based on their responsiveness to the treatment after 48 h, ZAR and CG lines were selected to investigate the molecular mechanism underlying the reduced cell migration observed compared to the untreated control.

**Figure 6:**
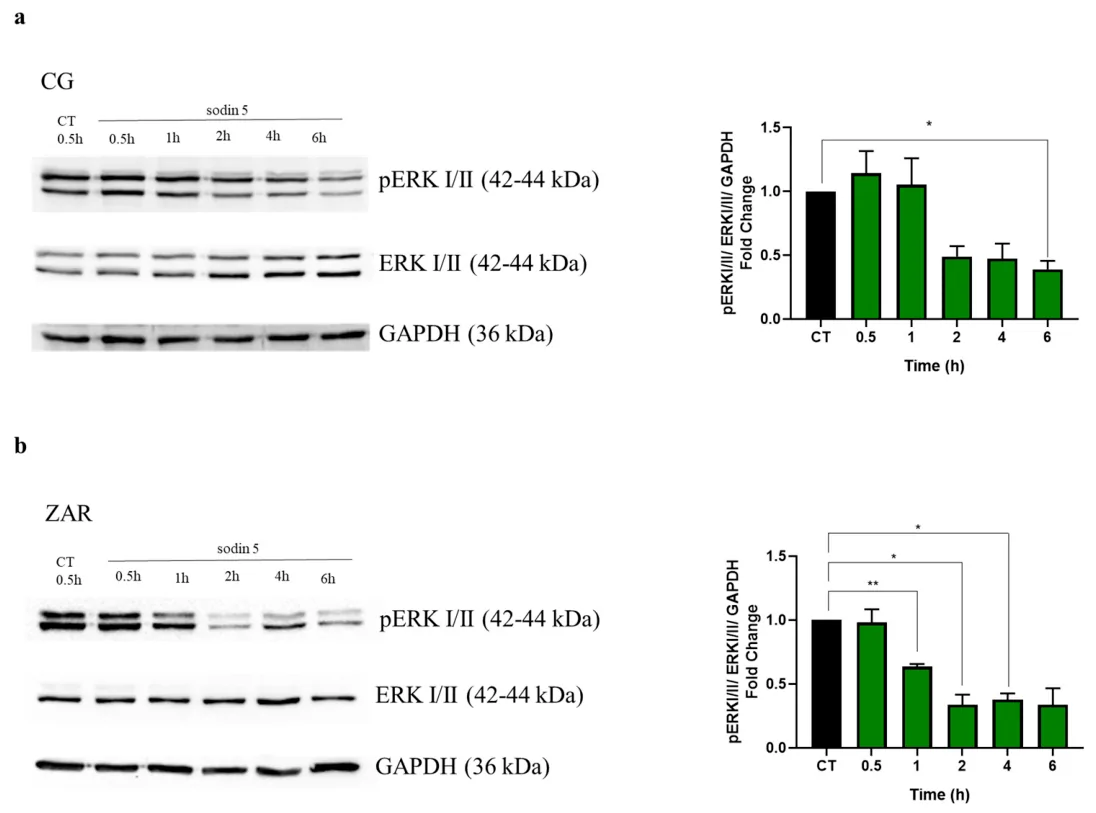
Western blot analysis of p-ERK I/II and ERK I/II proteins in the patient-derived CG (**a**) and ZAR (**b**) cell lines treated with 1.0 μM sodin 5 for 30 min, 1, 2, 4, and 6 h. GAPDH was used as a normalization control. The graph also shows the densitometric ratio of total p-ERK to total ERK in a bar chart: the untreated control (CT) is shown in black, while the treatments at different time points are in green. Data were analyzed using GraphPad Prism and are reported as mean ± SEM of three independent experiments (*n* = 3). Statistical analysis was performed using a one-way ANOVA followed by Dunnett’s multiple comparisons test: * *p* values < 0.05, ** *p* value 0.001 to 0.01 (very significant).

After 48 h of starvation in DMEM supplemented with 0.5% FBS, cells were treated with 1.0 µM sodin 5 and incubated for 30 min, 1, 2, 4, and 6 h. For each sample, 15 μg of total protein was loaded on a 12% polyacrylamide gel and transferred onto a PVDF membrane. Following overnight incubation with antibodies against ERK I/II and p-ERK I/II proteins, Western blot analysis revealed a reduction in p-ERK I/II protein levels relative to GAPDH expression (Figure 6). The decrease in p-ERK protein expression was already apparent 2 h after treatment, reaching its maximum reduction after 6 h in both cell lines.

**Figure 7:**
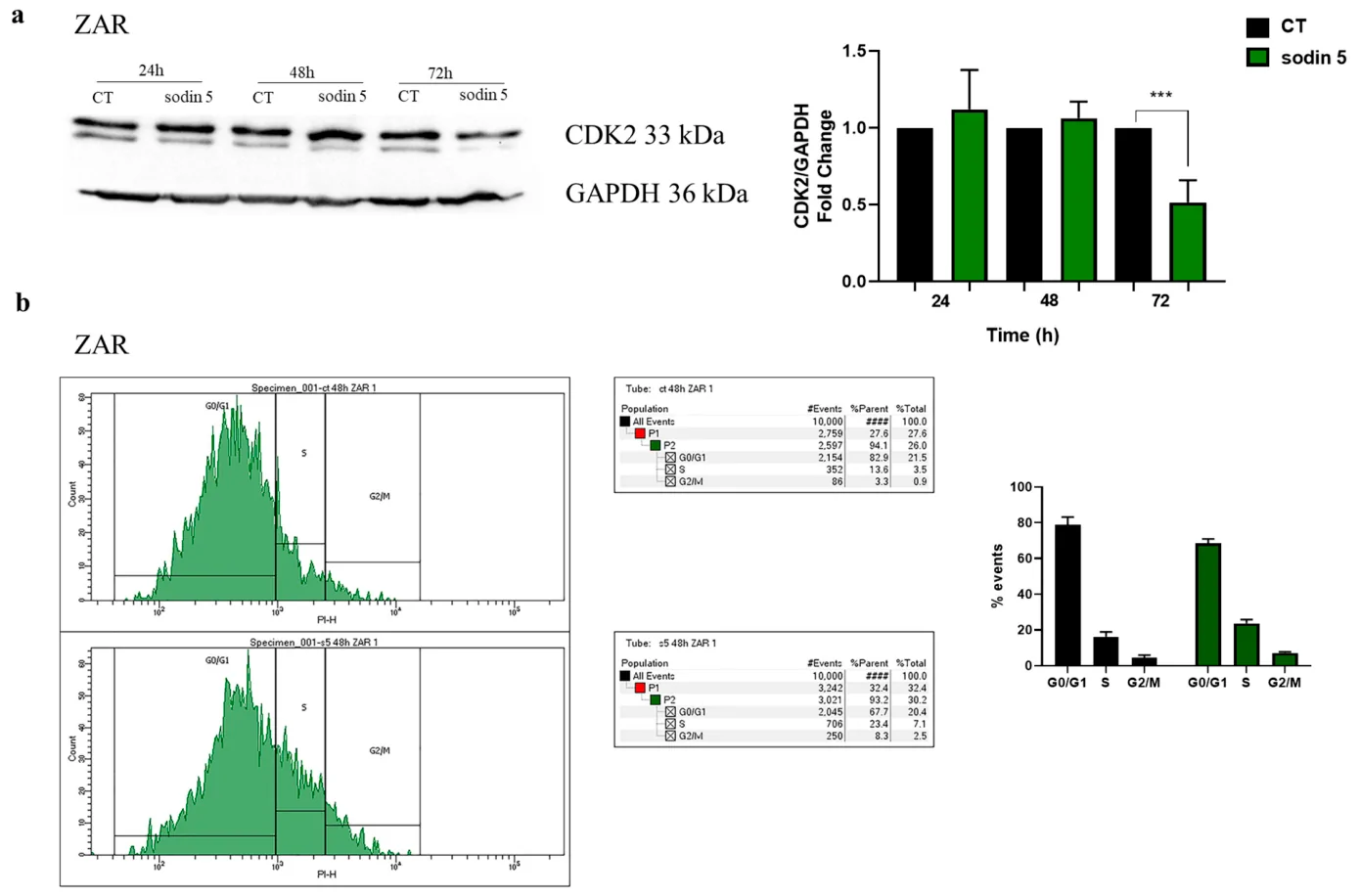
Western blot analysis of CDK2 protein in the patient-derived ZAR cell lines treated with 1.0 μM sodin 5 for 24, 48, and 72 h (**a**). Representative images of flow cytometric analysis of untreated ZAR cells (CT) and cells treated with 1.0 µM sodin 5 for 48 h (**b**). Data were analyzed using GraphPad Prism and are reported as mean ± SEM of three independent experiments (*n* = 3). Statistical analysis was performed using a one-way ANOVA followed by Tukey’s multiple comparisons test: *** *p* value 0.0005 (very significant).

To elucidate the molecular mechanism responsible for the inhibition of GBM cell growth, the expression levels of one of the key cell cycle regulatory proteins were determined by using Western blot analysis. In detail, long-term treatment of ZAR cells with 1.0 µM sodin 5 induced a significant reduction in CDK2 protein expression after 72 h of treatment (Figure 7a). Furthermore, cell cycle analysis using propidium iodide incorporation revealed that ZAR cells undergo G2/M phase arrest following 48 h of treatment with 1.0 µM sodin 5 (Figure 7b). These data will allow for future investigation of the proteins involved in this cell cycle phase.

### 2.5. PNAG Activity and Structural Features of Sodin 5 at Different Acidic pH Conditions

**Figure 8:**
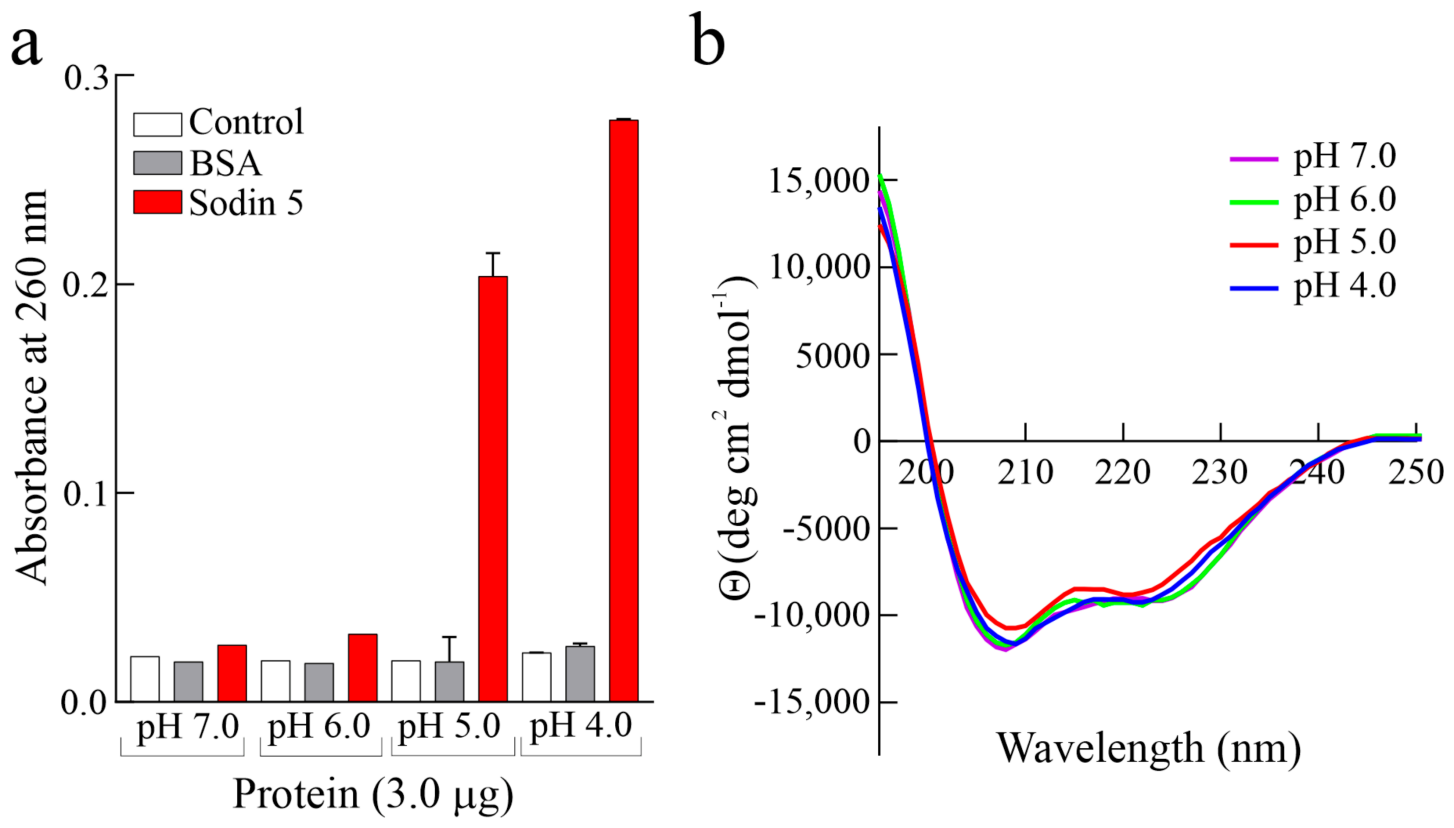
Stability of sodin 5 at different pHs. (**a**) Polynucleotide:adenosine glycosylase (PNGA) activity of BSA (negative controls) or sodin 5 assayed on ssDNA measured at different tested pHs. Proteins (3.0 μg) were assayed on salmon sperm DNA as described in the Materials and Methods Section 4.10. Data represent the mean of three biological replicates, each including three technical replicates ± SD. (**b**) Far-UV CD spectra of sodin 5 (0.15 mg mL^−1^) at different tested pHs.

Given the acidic shift observed in the glioma microenvironment [32], we investigated the enzymatic activity of sodin 5 across decreasing pH values by monitoring its PNAG activity. As shown in Figure 8a, the decrease in pH was associated with a significant increase in the PNAG activity of sodin 5. Interestingly, this trend agrees with previous findings obtained with quinoin, a type-1 RIP isolated from *Chenopodium quinoa* L. seeds [33]. Thus, this experimental evidence suggests that sodin 5 could effectively inhibit cell proliferation in acidic tumor microenvironments, such as those found in gliomas [32], with a potentially enhanced activity under low-pH conditions.

Furthermore, considering the increased enzymatic activity detected under acidic conditions, far-UV CD spectroscopy was employed to assess whether this enhancement could be attributed to pH-induced conformational changes affecting the secondary structure of sodin 5. However, decreasing the pH did not result in any significant spectral changes. Indeed, the CD spectra displayed an almost complete overlap across the tested pH range (Figure 8b), indicating that the protein structure remains substantially unaltered (Table S1). Therefore, the enhanced H^+^ concentration is unlikely to affect the overall secondary structure content of sodin 5, as indicated by the CD data, but it may induce subtle changes in intramolecular non-covalent interactions within the enzyme or within the enzyme–substrate (E-S) complex, thereby promoting PNAG activity at acidic pH [33].

## 3. Discussion

Despite attempts to improve outcomes for newly diagnosed patients, an effective treatment for GBM remains unresolved. In this context, we tested sodin 5, a novel type-1 RIP extracted from *S. soda* seeds, on the continuous glioblastoma cell line U87MG and on three patient-derived primary GBM cell lines: CG, ZAR, and NULU. Primary GBM lines represent an excellent in vitro study system as they closely retain the genetic and histological characteristics of the original tumors [31]. Preliminary experiments to evaluate the IC_50_ of sodin 5 revealed high toxicity in GBM cells across 24, 48, and 72 h of treatment at concentrations ranging from 0.01 to 1.0 µM.

The cytotoxic effect of sodin 5 was also evaluated on primary cell lines, revealing a lower toxicity profile compared to established lines. Treatment of U87MG with 0.1 µM sodin 5 resulted in a reduction in cell growth after 24 h, reaching a growth inhibition of approximately 50% after 72 h of induction. Conversely, primary lines exhibited a weaker inhibitory response to the treatment with 0.1 µM sodin 5; therefore, a higher concentration of 1.0 µM was used on the CG, ZAR, and NULU primary lines. Standard conventional GBM cell lines (e.g., U87MG) are known to be genetically distant from primary tumors due to the high number of passages in culture [31], which leads to a loss of the heterogeneity of GBM cells. Consequently, we chose to investigate the effect of sodin 5 on the patient-derived CG, ZAR, and NULU cell lines that maintain greater similarity to the tumor of origin and serve as a more reliable model for studying GBM [31].

The impact of sodin 5 on the migration of both patient-derived cell lines (CG, ZAR, and NULU) and the continuous glioblastoma cell line (U87MG) was assessed using a Trans-well migration assay and compared to the untreated controls. When exposed to a chemotactic gradient triggered by FBS stimulation, all the examined lines exhibited a decrease in cell migration upon sodin 5 treatment, as shown in Figure 4. Specifically, after 48 h of exposure to 0.1 μM sodin 5, the percentage of migrated U87MG cells was remarkably reduced by approximately 20% compared to 40% in the control group (Figure 4a). Comparable results were obtained for the primary lines; daily induction with 1.0 μM sodin 5 for 48 h led to a 20% reduction in migration (Figure 4b) for CG, while ZAR and NULU showed a decrease of approximately 15% (Figure 4c,d), relative to their respective controls. The coordinated action of various cytoskeletal networks tightly regulates cellular dynamics and migration. Alterations in the cytoskeleton of tumor cells are closely linked to the acquisition of an invasive phenotype and the loss of tissue integrity. For instance, elevated expression of the intermediate filament protein vimentin is associated with the metastatic potential in epithelial malignancies. Nevertheless, the precise mechanisms by which vimentin influences cell motility remain largely elusive [34]. In this work, we have demonstrated that treatment with sodin 5 decreases glioblastoma cell migration, which correlates with a concomitant reduction in vimentin expression, thereby indicating a direct involvement of this protein in the impaired movement of the treated cells. The primary CG cell line showed the highest sensitivity to sodin 5 treatment, prompting further investigation into the molecular mechanisms underlying the reduced migration compared to controls. The proteins ERK (Extracellular Signal-Regulated Kinases) and p-ERK (Phospho-Extracellular Signal-Regulated Kinases) play fundamental, albeit distinct, roles in regulating cell migration, a process essential for development, immune response, and tumor metastasis. In particular, the MAPK ERK I/II pathway is a key regulator of cell motility: once activated via phosphorylation (p-ERK), it controls actin cytoskeleton remodeling and focal adhesion dynamics, allowing cells to form membrane protrusions and migrate coordinately through the extracellular space. After 48 h of serum starvation, Western blot analysis of CG and ZAR cells treated with 1.0 µM sodin 5 showed a decrease in the expression of p-ERK I/II. This effect was evident particularly after 2 h, with the maximum reduction observed at 6 h, indicating a significant impact of impaired ERK phosphorylation on cell motility (Figure 5). Furthermore, it cannot be excluded that the decrease in ERK phosphorylation may also drive the attenuation of proliferative signals, thereby leading to the inhibition of growth in glioblastoma cells treated with sodin 5. ERK1/2 phosphorylation (p-ERK1/2) is a key activator of cellular proliferation, and its inhibition is a well-recognized mechanism for halting GBM cell growth [35,36]. Components and regulators of the ERK pathway are promising therapeutic targets for cancer, with MEK and BRAF inhibitors currently in clinical use. Although ERK1/2 kinases can induce tumor cell death under specific conditions, the mechanisms underlying ERK1/2-mediated apoptosis remain poorly understood [37].

In this context, sodin 5 could represent a novel therapeutic candidate for glioblastoma, given its ability to modulate ERK1/2 signaling and consequently inhibit tumor cell proliferation and migration. The preliminary flow cytometry analysis showed a redistribution of the cell population following treatment with sodin 5, highlighting that at 72 h an increase in the population in the G2/M phase occurs, suggesting that the sodin 5-dependent proliferative block may also be based on a component of the cell cycle block. In conclusion, our results suggest that sodin 5—a naturally occurring toxin—could be used in the future as an adjuvant agent in combination with TMZ (the standard chemotherapeutic agent for glioblastoma). Following engineering via conjugation with nanocarriers or specific antibodies, this approach could enhance the efficacy of standard treatment by modulating key signaling pathways to inhibit tumor growth. However, future in vivo studies will be required to confirm the validity of this strategy and verify that the limitations associated with systemic toxicity can be overcome.

## 4. Materials and Methods

### 4.1. Materials

All the chemicals for chromatography were purchased or obtained as previously reported [23]. Single-stranded salmon sperm DNA was obtained from Sigma-Aldrich Solutions (Merck Life Science S.r.l., Milan, Italy). U87MG cells were obtained from ATCC (Manassas, VA, USA). All other reagents and chemicals were of analytical grade. Further details for specific reagents or enzymes are reported in the respective paragraphs below.

### 4.2. Sodin 5 Purification

Native sodin 5 was purified from *S. soda* seeds as previously described by Landi et al. [23]. Briefly, the crude protein extract from the seeds was fractionated based on solubility at pH 4.0. The soluble basic protein fraction was then subjected to three sequential chromatographic steps: (i) stepwise cation-exchange chromatography; (ii) gel-filtration chromatography; and (iii) cation-exchange chromatography eluted with a linear gradient of NaCl. Fractions corresponding to purified sodin 5 were pooled, dialyzed against water, freeze-dried, and stored at −20 °C until use.

Protein concentration was determined using a Pierce BCA Protein Assay Kit (Life Technologies Italia Fil., Monza, Italy).

### 4.3. Cell Culture

U87MG cells were obtained from ATCC (Manassas, VA, USA), while primary human GBM cell lines (CG, NULU, and ZAR) were derived from brain biopsies of Neuromed neurosurgery patients who provided informed consent to participate in the study. The use of these primary cell lines as a model of GBM heterogeneity was approved by the ethics committee on 27 February 2020 and registered on ClinicalTrials.gov (NCT04180046). The GBM cell lines used in subsequent experiments were previously characterized, as reported by Oliva et al. (2021) [31]. All GBM cells were cultured in Dulbecco’s Modified Eagle’s Medium (DMEM) (GIBCO, Waltham, MA, USA) supplemented with 10% fetal bovine serum (FBS, GIBCO, Waltham, MA, USA), 2 mmol/L glutamine, 100 IU/mL penicillin, and 100 µg/mL streptomycin (GIBCO, Waltham, MA, USA) and maintained at 37 °C in a humidified atmosphere containing 5% CO_2_.

### 4.4. MTT for IC50 Determination of Sodin 5

The IC_50_ values of sodin 5 were determined in human continuous U87MG cells and primary CG cells. Briefly, cells were seeded in 96-well plates at a density of 5 × 10^3^ cells per well, treated once with increasing concentrations of sodin 5 (0.0001, 0.001, 0.01, 0.1, 1.0, and 2.0 µM) and then allowed to grow for 24, 48, and 72 h. The MTT assay was performed by adding 5.0 mg/mL of Thiazolyl Blue Tetrazolium Bromide (Sigma-Aldrich Solutions, St. Louis, Missouri, USA) to 100 µL of cells cultured in DMEM with 10% FBS. The formazan crystals were dissolved with 0.4% HCl in isopropanol, and the absorbance was measured at 595 nm with a plate-reading spectrophotometer. IC_50_ values for each condition were calculated by using nonlinear regression analysis (curve fit) applying the log(inhibitor) vs. normalized response, using GraphPad Prism 8 software (GraphPad Software Inc., San Diego, CA, USA).

### 4.5. Proliferation Assay

To assess the effect of sodin 5 on GBM cell proliferation, U87MG, NULU, CG, and ZAR cells were seeded in 48-well plates at a density of 1 × 10^4^ cells per well in DMEM supplemented with 10% FBS and incubated at 37 °C in a 5% CO_2_ atmosphere. U87MG cells were treated with sodin 5 at concentrations of 0.01 and 0.1 µM, whereas NULU, CG, and ZAR cells received 0.1 and 1.0 µM, with daily treatment administration. Cell proliferation was evaluated at 24, 48, and 72 h by performing cell counts using a Burker chamber. Growth curves were generated using GraphPad Prism 8 software (GraphPad Software Inc., San Diego, CA, USA). Data are reported as mean ± SEM of three individual experiments (*n* = 3): one-way ANOVA test, Tukey’s multiple comparisons test. According to GraphPad Prism, * *p* values < 0.05, ** *p* value 0.001 to 0.01 (very significant), **** *p* value < 0.0001 (extremely significant). For each treatment and each experimental time point, images were acquired using an EVOS FL microscope (Life Technologies, Carlsbad, CA, USA) at 10× magnification.

### 4.6. Trans-Well Migration Assay

For U87MG, NULU, CG, and ZAR, approximately 2 × 10^5^ cells per well were seeded in serum-free DMEM in the upper chamber of the Trans-well insert, while in the lower chamber were added 2.0 mL of FBS. For each cell line, two experimental conditions were set up: control and sodin 5-treated (0.1 µM for U87MG or 1.0 µM for NULU, CG, and ZAR); both conditions were analyzed at a single time point of 48 h. Cells adhering to the membrane were fixed with 75% ethanol and stained with Cresyl violet (DiaPath, Martinengo, Lombardy, Italy). Finally, for each slide, images were captured from five different microscopic fields at 100× magnification to assess cell migration across the membrane (Microscope Axiophot 2 Zeiss, Oberkochen, Germany). Data were analyzed using GraphPad Prism and are reported as mean ± SEM of three independent experiments (*n* = 3). Statistical significance was determined using an unpaired *t*-test: * *p* values < 0.05; ** *p* value 0.001 to 0.01 (very significant); **** *p* value < 0.0001 (extremely significant).

### 4.7. Immunofluorescence

ZAR cells (1 × 10^4^) were plated in 8-well chamber slides in DMEM with 10% FBS, treated with sodin 5 1.0 μM for 24 and 48 h, and the morphological changes induced by treatment were assessed by immunofluorescence staining for the cytoskeleton protein vimentin. In detail, after 24 and 48 h treatment, ZAR cells were fixed in 4% formalin (DiaPath, Martinengo, Lombardy, Italy) for 20 min and permeabilized with 0.1% Triton (Invitrogen, Carlsbad, CA, USA) for 30 min. After blocking with 10% specific serum, cells were incubated with anti-vimentin (prediluted; Roche diagnostic, 1:200, Abcam, Cambridge, MA, USA) overnight at 4 °C. After washing with 0.025% PBS-Tween-20, cells were incubated with secondary antibody anti-mouse fluorescein (1:100; Vector, Stuttgart, Germany) in 2% serum for 1 h at room temperature. The slides were counterstained with DAPI Mounting Medium (Sigma-Aldrich Solutions, St Louis, MO, USA) for nuclei detection and analyzed with a fluorescence microscope at 40× magnification. Data were analyzed with GraphPad Prism 8 and represent the mean of 12 different fields ± SD. Statistical significance was determined using an unpaired *t*-test: **** *p* value < 0.0001 (extremely significant).

### 4.8. Western Blot Analysis

CG and ZAR cells were seeded at a density of 5 × 10^5^ per 60-mm diameter dish in DMEM supplemented with 10% FBS and incubated at 37 °C with 5% CO_2._ The medium was then replaced with DMEM with 0.5% FBS for 48 h. Cells were treated with 1.0 µM sodin 5, and untreated cells were used as a control. For pERKI-II and ERKI-II analysis, cells were collected after 30 min, 1, 2, 4, and 6 h, and the proteins were extracted using lysis buffer (10 mM Tris-HCl, 1.0 mM EDTA, 150 mM NaCl, 1% Triton X-100, 1.0 mM NaF, 1.0 mM Na_4_P2O_7_, 1 mM Na_3_VO_4_, and 1× protease and phosphatase inhibitors). 20 μg of total proteins were separated on SDS-PAGE and then transferred to PVDF membranes. The membranes were incubated for 1 h at room temperature in 5% BSA (Bovine serum albumin, Sigma-Aldrich, St Louis, MO, USA) diluted in a solution of TBS 1× and Tween-20 (TBS-T) and then incubated overnight at 4 °C with anti-pERKI-II (1:2000 Elabscience reagents, Ltd., Wuhan, Hubei, China), anti-ERK I-II (1:2000 Elabscience reagents, Ltd., Wuhan, Hubei, China), and then incubated with anti-rabbit or anti-mouse HRP secondary antibody (1:3000 Cell Signaling, Danvers, MA, USA) for 1 h at room temperature. For CDK2 analysis, cells were collected after 24, 48, and 72 h, and the proteins were extracted using lysis buffer (10 mM Tris-HCl, 1.0 mM EDTA, 150 mM NaCl, 1% Triton X-100, 1.0 mM NaF, 1.0 mM Na_4_P2O_7_, 1 mM Na_3_VO_4_, and 1× protease and phosphatase inhibitors). 20 μg of total proteins were separated on SDS-PAGE and then transferred to PVDF membranes. The membranes were incubated for 1 h at room temperature in 5% BSA (Bovine serum albumin, Sigma-Aldrich, St Louis, MO, USA) diluted in a solution of TBS 1× and Tween-20 (TBS-T), and then incubated overnight at 4 °C with anti-CDK2 (1:1000 Cell Signaling) antibody and then incubated with anti-rabbit HRP secondary antibody (1:3000 Cell Signaling, Danvers, MA, USA) for 1 h at room temperature. Following three washes, the antigen/antibody proteins were detected by chemiluminescence using ECL Western blotting (Bio-Rad, Hercules, CA, USA). The signals were detected by a digital scanner and quantified with iBright Analysis Software V5.4.0.exe (ThermoFisher Scientific, Waltham, MA, USA). For protein normalization, each membrane was then incubated with a mouse monoclonal antibody anti-GAPDH (1:2500 Abcam, Cambridge, Cambridgeshire, UK). Data were analyzed using GraphPad Prism and are reported as mean ± SEM of three independent experiments (*n* = 3). Statistical analysis was performed using a one-way ANOVA followed by Dunnett’s multiple comparisons test: * *p* values < 0.05, ** *p* value 0.001 to 0.01 (very significant) or by Tukey’s multiple comparisons test: *** *p* value 0.0005 (very significant).

### 4.9. Cell Cycle Analysis by Flow Cytometry (FACS)

ZAR cells were seeded in DMEM supplemented with 0.5% FBS (4 × 10^5^ cells/well) for 48 h and treated with 1.0 µM sodin 5 for 24, 48, and 72 h. Untreated cells served as a control. Following treatment, cells were washed in sample buffer (0.1% glucose in HBSS without Ca^2+^ and Mg^2+^), resuspended in the same buffer, and fixed in ice-cold 70% ethanol overnight at 4 °C. After centrifugation, the cell pellets were incubated in a propidium iodide (PI) staining solution containing 50 µg/mL of PI (BD Pharmigen, San Diego, CA, USA) for 30 min at room temperature and analyzed using a flow cytometer (BD FACSCelesta™ instrument, Franklin Lakes, NJ, USA).

### 4.10. Polynucleotide:Adenosine Glycosylase (PNAG) of Sodin 5 Activity on Salmon Sperm DNA

PNAG activity of sodin 5 was assayed on single-stranded DNA (ssDNA) from salmon testes. Equal amounts of sodin 5 [0.1 nmol (~3.0 µg; 0.34 µM) were incubated with 100 µg of ssDNA (final concentration 0.33 mg mL^−1^) at 30 °C for 1 h in 10 mM buffers at different pHs (Na-phosphate at pH 7.0 or pH 6.0, Na-acetate at pH 5.0 or pH 4.0) (final volume 0.3 mL). The enzyme: substrate ratio was about 1:33; *v*:*v*. Following incubation, DNA was precipitated overnight with 2.5 volumes of cold ethanol in the presence of Na-acetate 0.14 M pH 5.2 (0.1 volume) at −80 °C and centrifuged at 18,000 *g* for 30 min at 4.0 °C. Adenine released was determined in the supernatants spectrophotometrically at 260 nm. The same amount of BSA (3.0 µg) was used as a negative control. Data represent the mean of three biological replicates, each including three technical replicates ± SD.

### 4.11. Circular Dichroism of Sodin 5 at Different pHs

Far-UV circular dichroism (CD) spectra, used to estimate protein secondary structure, were recorded at 25 °C on a Jasco J-815 dichrograph [Jasco Europe, Cremella (LC) Italy]. Measurements were performed using a 0.1 cm path-length quartz cuvette with a protein concentration of 0.15 mg/mL (~5.0 µM) in 10 mM buffers at different pHs: Na-phosphate (pH 7.0 and 6.0) and Na-acetate (pH 5.0 and 4.0). Background signals were subtracted from all samples.

## Data Availability

The original contributions presented in this study are included in the article/Supplementary Material. Further inquiries can be directed to the corresponding authors.

## Supplementary Materials

The following supporting information can be downloaded at: https://www.mdpi.com/article/10.3390/toxins18100441/s1, Table S1. Estimated secondary-structure content (%) of sodin 5 at different pH values, based on circular dichroism spectra.

## Abbreviations

The following abbreviations are used in this manuscript:

RIP	Ribosome-inactivating protein
rRNA	Ribosomal ribonucleic acid
SRL	Sarcin–Ricin Loop
PNAG	Polynucleotide:adenosine glycosylases
RNA	Ribonucleic acid
DNA	Deoxyribonucleic acid
GBM	Glioblastoma
MGMT	O6-Methylguanine methyltransferase
TMZ	Temozolomide
CD	Circular dichroism
IC50	Inhibitory concentration 50
ERK	Extracellular Signal-Regulated Kinases
pERK	Phospho-Extracellular Signal-Regulated Kinases
DMEM	Dulbecco’s Modified Eagle’s Medium
FBS	Fetal bovine serum
PVDF	Polyvinylidene fluoride
CDK2	Cyclin-Dependent Kinase 2
MEK	Methyl Ethyl Ketone
ATCC	American Type Culture Collection
MTT	3-(4,5-dimethylthiazol-2-yl)-2,5-diphenyltetrazolium bromide
HCl	Hydrochloric acid
NaCl	Sodium chloride
EDTA	Ethylenediaminetetraacetic acid
SDS-PAGE	Sodium Dodecyl Sulfate Polyacrylamide Gel Electrophoresis
PI	Propidium Iodite
FACS	Fluorescence-Activated Cell Sorter
BRAF	B-rapidly accelerated fibrosarcoma
