# Supplementary material for: Effects of Sodin 5 on the Proliferation and Migration of Human Glioblastoma Cells
Source: Toxins (Basel). 2026 Oct 13;18(10):441. doi: 10.3390/toxins18100441 (PMC13709611; doi:10.3390/toxins18100441)
Supplement: Supplementary file 1 [file toxins-18-00441-s001.zip › toxins-4466327-supplementary.pdf]

Supplementary Materials: Effects of Sodin 5 on the Proliferation and Migration of Human Glioblastoma Cells

Veronica Russo [1,†], Sara Ragucci [2,†], Miriam Russo [1], Maria Antonietta Oliva [1], Nicola Landi [2,3], Antimo Di Maro [2,*] and Antonietta Arcella [1,*]

[1] IRCCS Istituto Neurologico Mediterraneo NEUROMED, Via Atinense 18, 86077 Pozzilli, IS, Italy; veronica.2306@hotmail.it (V.R.); miriamrusso39@gmail.com (M.R.); mariaantonietta.oliva@neuromed.it (M.A.O.)

[2] Department of Environmental, Biological and Pharmaceutical Sciences and Technologies (DiSTABiF), University of Campania "Luigi Vanvitelli", Via Vivaldi 43, 81100 Caserta, CE, Italy; sara.ragucci@unicampania.it (S.R.); nicola.landi@cnr.it (N.L.)

[3] Institute of Crystallography, National Research Council, Via Vivaldi 43, 81100 Caserta, CE, Italy

* Correspondence: antimo.dimaro@unicampania.it (A.D.M.); arcella@neuromed.it (A.A.)

† These authors contributed equally to this work.

Table S1. Estimated secondary-structure content (%) of sodin 5 at different pH values, based on circular dichroism spectra.

	α-helix	β-strand	β-turn	Other
pH 7.0	27.6±1.65	14.1±0.68	13.9±0.79	44.4±2.17
pH 6.0	28.1±1.38	15.5±0.91	13.5±0.66	43.2±2.29
pH 5.0	27.8±1.19	16.4±0.75	13.7±0.83	41.3±2.07
pH 4.0	28.9±1.56	16.1±0.92	12.9±0.61	42.8±2.44
